# Trend analysis and prediction of injury death in Xi’an city, China, 2005-2020

**DOI:** 10.1186/s13690-022-00988-y

**Published:** 2022-11-19

**Authors:** Xiao-Yu Zhang, Lin-Lin Ma, Ning Chen, Dan-Dan Wu, Yu-Xiang Yan

**Affiliations:** 1 Department of Chronic Disease Management, Xi’an Center for Disease Control and Prevention, Xi’an, 710054 China; 2 Department of Epidemiology and Biostatistics, School of Public Health, Capital Medical University, Beijing, 100069 China

**Keywords:** Injury, Mortality, Rank, Distribution, Trend, Prediction

## Abstract

**Background:**

Injury is an important cause of death in China. In the present study, we systematically analyzed the epidemiological characteristics and trends of injury death in Xi’an residents from 2005 to 2020.

**Methods:**

Data on injury deaths from 2005 to 2020 were obtained from the “Xi’an Center for Disease Control and Prevention”, injury deaths were classified according to the International Classification Disease-10th Revision (ICD-10). The data were stratified by gender, age groups, injury types, and then overall and type-specific injury mortality rates were estimated. Joinpoint regression analysis was conducted to estimate annual percent change (APC). The grey interval predicting method was used to predict the future characteristics of injury deaths in Xi’an city.

**Results:**

From 2005 to 2020, injury caused 32,596 deaths (5.79% of all deaths; 35.71/100000 population). Injury mortality rates were higher among males than females. Motor vehicle traffic accidents were the commonest injury type. The highest injury mortality rates were in those aged 85 years or older. Overall, Joinpoint regression analysis revealed that injury mortality had significantly (*p* < 0.05) decreasing trends. GM (1,1) model estimated that injury mortality will be on a declining curve.

**Conclusions:**

Motor vehicle traffic accidents, transport accidents other than motor vehicles, unintentional falls, suicide, and accidental poisoning are the main causes of injury. The injury death rate is projected to decline over the next decade.

**Supplementary Information:**

The online version contains supplementary material available at 10.1186/s13690-022-00988-y.

## Background

International (homicide and suicide) and unintentional injuries are major causes of death in many countries, and they can stymie society’s economic progress through direct and indirect costs [1]. Injury is now considered a worldwide epidemic and affects families and communities in dramatic ways [2]. Every day, almost 16,000 individuals die from injuries all around the world. In China, injuries are expected to claim 700,000 lives per year, accounting for 9% of all-cause deaths and ranking as the fifth-greatest cause of death [3]. According to indirect estimates from the World Health Organization (WHO) and the Global Burden of Diseases Study (GBD), unintentional injuries cause 3.9 million fatalities globally, with 90% of those deaths occurring in low- and middle-income countries [4]. Road traffic injuries falls, drowning, poisoning, and burns account for the majority of these deaths [4]. The national impact, particularly on healthcare and insurance expenditures, is enormous. The effects on communities, on the other hand, are massive and multifaceted. Communities are affected not only by the emotional and psychological consequences of injury-related deaths but also by the economic and social consequences of injury-related disabilities. Job loss, injury care, rehabilitation costs, family disruption, medical care visits, insurance claims, and marital troubles all carry enormous economic and social costs [2]. In terms of disability-adjusted life years lost, traumatic injuries account for 8.5% of mortality and 12% of the worldwide burden of diseases [5]. Road traffic injuries are responsible for about a quarter of all deaths from external causes worldwide, with an estimated 1.25 million deaths in 2013, or 24,1 deaths per 100,000 people [6–8]. The specific sorts of injuries that occur, as well as their risk factors, occurrences, and effects, vary between societies and even within subgroups within the same society, making detailed studies of injury patterns essential for proposing effective remedies [9]. The world health organization in the global road safety situation (2018) report [10], the damage caused by road traffic accident death toll continued to climb, the death toll at about 1.35 million people a year, and the road traffic injuries is 5 ~ 29 children first cause of death among young people. If it is not the current road traffic accidents that take corresponding prevention measures, by 2030 it will be the fifth leading cause of death globally.

In their influential study, Kim et al. first proposed Joinpoint regression model in 1998 [11]. The core idea of this model is to establish segmented regression according to the time characteristics of disease distribution, divide the study time into different intervals through several connection points, and conduct trend fitting and optimization for each interval. Furthermore, the characteristics of different interval-specific disease changes in the global time range were evaluated in more detail. This method is particularly useful in the research field of mortality trends.

The grey series prediction model GM (1,1) is a common analysis method based on the time-series data, which transforms the irregular original sequence, establishes the regular regression equation of the generated sequence, and uses the equation to predict the dynamic development trend of the disease. GM (1,1) often be selected for its reliability and validity. A major advantage of GM (1,1) is that it can be used for recent, short-term, and long-term predictions.

A prefecture, the provincial capital, a sub-provincial city, and a megalopolis, Xi’an is the core city of Guan Zhong Plain urban agglomeration, an important city in the province of Shanxi. The development of Xi’an is of special significance to Shanxi Province and northwest China, and its death situation is of representative significance. Injury is the fifth biggest cause of death and disability in Xi’an; it is also a prominent source of premature mortality and disability. To deeply understand the injury death status of permanent residents in Xi’an city and provide a scientific basis for formulating injury prevention and control measures, this paper analyzed the injury death data of permanent residents in Xi’an city, Shanxi Province from 2005 to 2020.

## Methods

### Data collection

Population-wide mortality data were collected from the “Xi’an Center for Disease Control and Prevention”, which includes the name, sex, age, death event, death time, death place, occupation, direct cause of death, root cause of death, etc. Population data from Xi’an Public Security Bureau. Chinese population data came from United Nations Population Division.

All methods used in this study were carried out in accordance with relevant guidelines and regulations. This study was approved by the Ethics Committees of Xi’an Center for Disease Control and Prevention (CDC) and Capital Medical University.

### Classification of injury deaths

Injury-related deaths were classified according to the *International Classification of Diseases, Tenth Revision (ICD-10)* [12]. The codes identified the fourteen major causes of death (V01-Y98): motor vehicle traffic accidents, motor vehicles outside of transportation accidents, accidental poisoning, unintentional falls, fires, accidents caused by natural environmental factors, drowning, accidents of mechanic asphyxia, batter to death, caused by the mechanical cutting and piercing tools of accident, electric shock, accidents, and other harmful effects, suicide, and homicide.

### Statistical analysis

The data was collected and analyzed using SPSS 26.0 and Excel 2019. Number of deaths, mortality, standardized mortality, constituent proportion, the rank of death cause, average number of years of life lost (AYLL), potential years of life lost rate (PYLLR), and the rank of life lost by injury are some of the statistical analysis indicators. To compare injury mortality data between various groups in different eras and with varied features, the χ^2^ test or *Fisher’s* exact probability test was utilized. *Fisher’s* exact probability test was used to compare the data of injury mortality between different groups in different periods and with different characteristics, with a level α = 0.05. The injury mortality is the ratio of total injury deaths during the year to the average annual total population, expressed as 100,000. Joinpoint Regression Program (V.4.7.0.0, National Cancer Institute, 2019) was used to analyze and test the time variation trend of injury mortality. With injury mortality as the dependent variable and the year as the independent variable, the linear regression model was fitted to analyze and the annual percent change (APC) was obtained. $APC=\left[\frac{y_{x+1}-{y}_x}{y_x}\right]\ast 100=\left({e}^{\beta_1}-1\right)\ast 100$. In the formula, y is the mortality rate, x is the year, and *β*_1_ is the regression coefficient. The *t*-test was adopted, and the statistical significance of APC was used as the criterion for trend judgment. This analysis’ results are classified as significant (*p* < 0.05), marginally significant (0.05 < *p* ≤ 0.10), or nonsignificant otherwise [13].

### Quality control method

The correctness of the cause-of-death codes extracted by the cause-of-death surveillance system was further checked before analyzing the data [14].

## Results

### Injury death profile

**Fig. 1:**
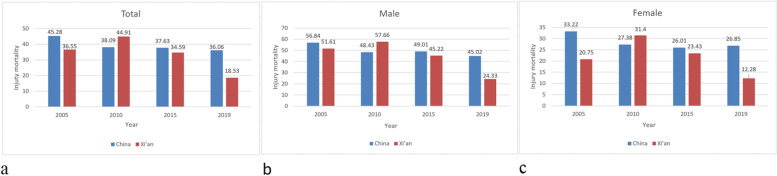
Comparison of injury mortality between in Xi’an and China

Table 1 presents an overview of injury death in Xi’an. During the 16 years, a total of 562,219 deaths were recorded in Xi’an. Injury-related deaths account for approximately 5.79% of all deaths due to all causes. The injury mortality of permanent residents in Xi’an city ranges from 15.56/100000 (2020) to 47.78/100000 (2011). The average injury mortality was 35.71/100000. Males tended to have higher injury mortality than females each year. (*P* < 0.05). Compared with nationwide, the injury mortality rates in Xi’an were significantly lower in 2005, 2015, 2019 and higher in 2010 (Fig. 1a-c Comparison of injury mortality between Xi’an and China).

**Table 1:** Injury death profile of permanent residents in Xi’an, 2005-2020 (N, 1/100000)

Year	Average Population	Total		Male		Female		***χ***^***2***^	***P*** value
		Death toll	Injury mortality	Death toll	Injury mortality	Death toll	Injury mortality		
2005	2,962,802	1083	36.55	783	51.61	300	20.75	192.944	< 0.05
2006	3,621,722	1387	38.21	984	53.95	403	22.32	357.276	< 0.05
2007	3,777,757	1782	47.17	1290	66.23	492	26.88	310.976	< 0.05
2008	4,310,677	1742	40.38	1252	56.15	490	23.51	284.356	< 0.05
2009	4,289,191	1995	46.35	1373	61.96	622	29.78	246.616	< 0.05
2010	4,358,204	1962	44.91	1296	57.66	666	31.40	167.695	< 0.05
2011	4,610,259	2203	47.78	1564	66.19	639	28.43	343.897	< 0.05
2012	4,959,830	1739	35.06	1194	46.98	545	22.54	211.239	< 0.05
2013	6,274,832	2523	40.21	1775	55.71	748	24.22	386.836	< 0.05
2014	6,985,866	2682	38.39	1869	52.08	813	23.93	366.528	< 0.05
2015	7,112,866	2460	34.59	1647	45.22	813	23.43	244.020	< 0.05
2016	8,249,323	2562	31.06	1710	40.38	852	21.22	243.569	< 0.05
2017	8,450,861	2968	35.12	1979	45.72	989	23.99	284.012	< 0.05
2018	9,616,700	2065	21.47	1446	29.42	619	13.31	279.218	< 0.05
2019	10,003,681	1854	18.53	1263	24.33	591	12.28	195.889	< 0.05
2020	10,213,704	1589	15.56	1111	20.99	478	9.71	208.558	< 0.05
**Total**	99,798,275	32,596	35.71	22,536	48.41	10,060	22.36	1100.203	< 0.05

### Analysis of life lost by injury

**Fig. 2:**
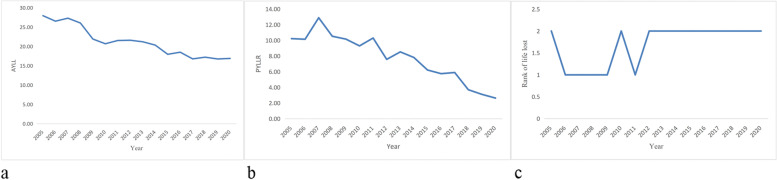
Analysis of life lost caused by injury in Xi’an

AYLL, PYLLR, and rank of life lost by injury from 2005 to 2020 were presented in Fig. 2a-c (Analysis of life lost caused by injury in Xi’an). The graph shows that there has been a decrease in AYLL and PYLLR caused by injury in Xi’an city. And the rank of life lost by injury has been in the top 2 for 16 years.

### Injury deaths by age group

During the 16 years, the injury mortality ranges from 158.67/100000 (10-year-old group) to 8249.32/100000(85-year-old group). As shown in Additional Table 1, in the age trend, the overall injury mortality decreased first and then increased from 15-year-old.

### Major injury deaths

Broad categories of external causes of death can be defined based on the nature of the causes of injuries, and include motor vehicle traffic accidents, motor vehicles outside of transportation accidents, accidental poisoning, unintentional falls, fires, accidents caused by natural environmental factors, drowning, accidents of mechanic asphyxia, batter to death, caused by the mechanical cutting and piercing tools of accident, electric shock, accidents and other harmful effects, suicide and homicide 14 categories. From 2005 to 2020, the top five causes of injury death among the permanent population in Xi’an were motor vehicle traffic accidents, transport accidents other than motor vehicles, unintentional falls, suicide, and accidental poisoning. The mean mortality rates were 140.04/100000 (24.41%), 116.67/100000 (20.34%), 95.86/100000 (16.54%), 58.67/100000 (10.23%) and 46.63/100000 (8.13%), respectively. The top five causes of injury death of different genders were similar to that of the general population (Additional Table 2).

### Ranks in different age groups

The main causes of injury death were also different among different ages (Additional Table 3). In the 0-year-old group, accidental mechanical asphyxia was the leading cause of injury fatality. Motor vehicle traffic accidents are the leading cause of injury mortality among persons aged 5 to 84, and they rank among the top three causes of injury death for people of all ages. Unintentional falls are the leading cause of death among people aged 85 and up, and the elderly are particularly vulnerable to them. Additional Fig. 1 provides the age distribution for the top five causes of injury mortality.

### Seasonal distribution of mortality of various injury types in Xi’an city

**Fig. 3:**
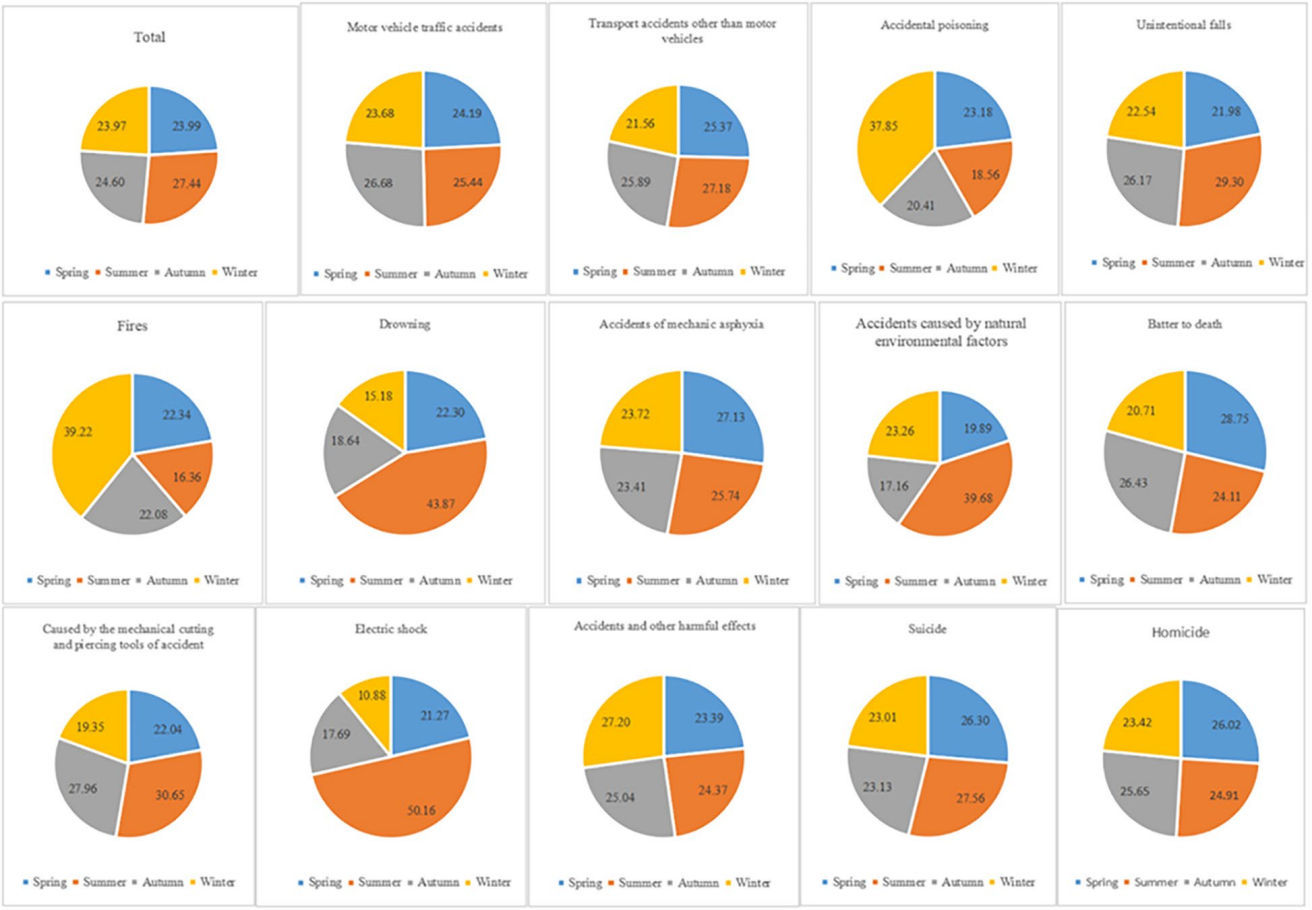
Seasonal distribution of injury mortality in Xi’an

As can be seen from Fig. 3 (Seasonal distribution of injury mortality in Xi’an), there was no significant difference between seasons in the overall injury. In terms of injury types, 43.87% of drowning accidents, 39.68% of accidents were caused by natural environment factors and 50.16% of electric shock accidents occurred in summer, accounting for a large proportion. The main injury types in winter were accidental poisoning (37.85%) and fire (39.22%).

### Location of injury deaths in Xi’an city

The study listed the locations of death events in all study samples and the corresponding constituent ratios, including hospital, emergency clinic, home or on the way to hospital, overseas and others, family inpatient ward and not quite clear. The ranking is based on respective fatality rates for each type of injury, as shown in Additionl Figs. 2 and 3. The majority of deaths occurred at home or on the way to hospital, accounting for 56.04% of the total. 21.15% of deaths occurred in hospitals, followed by Emergency clinics, overseas, others, and family inpatient wards.

### Occupational distribution of injury-related deaths in Xi’an city

The distribution of injury-related deaths was further analyzed according to occupation (Additional Figs. 4 and 5). Occupational classifications include technicist, office clerk, infants, preschool children or students, out of work, peasant, worker, and unknown or other workers. In the overall injury, the proportion of death caused by injury was the highest among peasants (62.28%), followed by the unemployed, workers, students, and office clerks, and the proportion of death caused by injury was the least among technicians.

### Trends in injury mortality for the top five causes of injury, 2005-2020

**Fig. 4:**
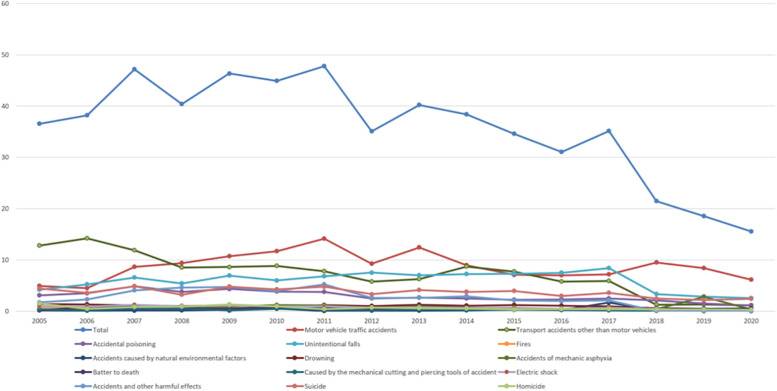
Trends in mortality rates by injury types in Xi’an

Figure 4 (Trends in mortality rates by injury types in Xi’an) shows the 16-year trend of death rates for each injury type. Joinpoint Regression Program (V.4.7.0.0, National Cancer Institute, 2019) was used to analyze the time series trend of the overall injury from 2005 to 2020 and the injury mortality of the top five injury causes (Table 2, Additional Tables 4, 5, 6, 7, 8 and 9). The result reflects a decreasing trend over 16 years (Overall, 2009-2017: APC = -5.0,2017-2020: APC = -22.3)

**Table 2:** Time series trends in overall injury mortality in Xi’an

Lower Endpoint	Upper Endpoint	APC	Lower CI	Upper CI	Test Statistic (t)	Prob > |t|
2005	2009	6.2	−3.2	16.5	1.5	0.173
2009	2017	−5.0	−8.6	−1.2	−3.0	0.017
2017	2020	−22.3	−32.8	−10.0	−4.0	0.004

### Prediction of injury mortality in Xi’an City, 2021-2030

The GM (1,1) model was established based on the injury death data from 2005 to 2020 to predict the injury death rate from 2021 to 2030. After model construction, a posterior error ratio C value is obtained, which is the residual variance/data variance. It is used to measure the fitting accuracy of the model, and the smaller the C value is, the better, generally less than 0.65. For mortality prediction, after calculation and selection, we found the best model of disease to predict its future mortality. It can be considered that the model fits well, the model error is relatively small, and the prediction result is relatively accurate. The overall injury death rate, motor vehicle traffic accident death rate, transportation accident death rate other than motor vehicle, suicide death rate, and accidental poisoning death rate were all qualified. In the model of unintentional falls mortality, whether total, male or female, the posterior error ratio C value was greater than 0.65 (0.7846, 0.6614, 0.9083), and grade accuracy was all unqualified. Therefore, data from 2005 to 2009 was removed, and the unintentional falls mortality rate was modeled and predicted again with the data from 2010 to 2020. The accuracy of the obtained model was qualified. The injury mortality is projected to continue decreasing in the future (Table 3, Additional Tables 10, 11, 12, 13 and 14).

**Table 3:** Overall injury mortality prediction in Xi’an

Year		Injury mortality	
	Total	Male	Female
2021	35.35	25.38	13.36
2022	34.16	22.67	12.22
2023	32.98	19.99	11.09
2024	31.82	17.37	9.96
2025	30.68	14.79	8.85
2026	29.55	12.25	7.76
2027	28.44	9.76	6.67
2028	27.35	7.31	5.60
2029	26.27	4.90	2.54
2030	25.21	2.54	3.48
**C value**	0.4884	0.1850	0.3395

## Discussion

In the present study, we systematically analyzed the epidemiological characteristics and trends of injury death in Xi’an residents from 2005 to 2020. There were 32,596 people who died of injuries during the 16 years, with annual injury mortality of 35.71/100000, lower than the injury mortality reported in the third National Inquest of Deaths (61.51/100,000) [15], ranking the fifth in the order of causes of death among permanent residents in Xi’an from 2005 to 2020. Studies both in China and other nations have shown that there are differences in mortality and patterns of injury in men and women [16, 17]. It can be seen that although the AYLL and PYLLR caused by injury in Xi’an are decreasing in recent years, the injury situation is still severe, and injury has become one of the important public health problems endangering the life safety of Xi’an residents.

The annual mortality rate in males is significantly higher than that of female mortality, which is consistent with other domestic results [18–22]. This discrepancy could be attributed to occupation. Their work may be risky, and their life and work pressure lead to psychological strains. Mental pressure is more likely too large to produce self-harm behavior. Males also exhibited stronger risk-taking behaviors than females [19]. Therefore, it is critical to improving male health education.

From 2005 to 2020, the top five causes of injury death among the permanent population in Xi’an were motor vehicle traffic accidents, transport accidents other than motor vehicles, unintentional falls, suicide, and accidental poisoning. The top five causes of injury are the same for both males and females. Motor vehicle traffic accidents were the leading cause of injury among permanent residents in Xi’an from 2005 to 2020. Therefore, the prevention of motor vehicle traffic accidents is the primary task of injury prevention in Xi’an city. The situation of death caused by traffic accidents for all ages is serious, and residents’ awareness of civilized driving and safe driving should be strengthened. Unintentional falls were the leading cause of death in the 85 and older group, and the elderly are the high-risk group for unintentional falls. Falls are commonly connected with severe morbidity and are a symptom of poor health and diminishing function [23]. In contrast to children, elderly individuals who fall are 10 times more likely to be hospitalized and 8 times more likely to die as a result of a fall [24]. Simultaneously, suicide is one of the leading causes of injury death, coming in fourth place. Suicide is a global public health epidemic that kills an estimated 800,000 people each year, with 85% of deaths happening in low- and middle-income nations. Suicides may be far more common than this figure, as they are frequently under-reported for a variety of societal, economic, and political reasons (Pattonet al., 2009 and Soron, 2018a) [25]. Suicide is often the result of comprehensive effects of many factors [26]. The mortality rate of male suicide is higher than that of female suicide. A possible explanation for this might be that men are facing greater employment and life pressure. Therefore, special attention should be paid to the male group when carrying out suicide prevention intervention, and targeted intervention measures should be taken.

Drowning, accidents caused by natural environmental factors, and electric shock mainly occur in summer (June, July, and August), which may be related to the extreme weather in summer. The main injury types in winter were accidental poisoning and fire. In winter (December, January, and February), gas combustion is not good to bring about toxic gas cannot be discharged in time, easy to produce poisoning. Inferior electrical appliances and combustible objects promote the cause of fire.

The occupational distribution of the death group of each injury type was similar, except that students had the second-highest mortality rate in drowning accidents. Government should strengthen peasant injury protection. Summer is the peak season for drowning accidents. According to incomplete figures from the National Health Commission and the Ministry of Public Security, 57,000 people die of drowning in China every year, among which children and teenagers account for 56% of the total, with an average of 88 children dying from drowning every day [27]. Therefore, drowning is known as “the number one killer of unnatural death of primary and secondary school students in China” [28].

Overall injury mortality was on the decline, following a similar pattern to the national and other areas [19, 27, 29–32]. The decrease in the injury mortality of residents in Xi’an indicates that the effect of injury prevention and control is relatively remarkable in recent years. It seems possible that the result is due to the improvement of injury prevention awareness and medical aid level of the local population. Time series trend analysis and grey model prediction results showed that the overall injury mortality and the top five injury mortality in Xi’an were on a downward trend from 2005 to 2020, and may continue to decline in 2021-2030. But the injury mortality situation is still very serious. The government should pay attention to injury-related deaths and take different prevention and control measures for different groups. The grey model utilized in this study, however, can only reflect the data’s regularity; it needs to be refined and evaluated in conjunction with the actual situation. To improve forecast accuracy, new data should be regularly integrated into the follow-up for rapid illness monitoring and dynamic model prediction.

Although the occurrence of injury will be affected by age, gender, race, injury type, injury location, injury location and other factors [33, 34], according to the trend of injury mortality in recent years, using accurate models to predict future mortality can well predict the future trend of injury events based on existing prevention and control measures, and provide a basis for the government to implement targeted injury prevention and control strategies, To better reduce the incidence of injury deaths in the future, which has good public health significance.

## Conclusions

Taken together, these results suggest that injury remains a leading cause of death in Xi’an. Motor vehicle traffic accidents, transport accidents other than motor vehicles, unintentional falls, suicide, and accidental poisoning are the main causes of injury and they should be the priorities of intervention, Over the next decade, injury death rates are predicted to decline. To reduce injury mortality in Xi’an, China, policies focusing on the main causes of death determined by research should be implemented.

## Supplementary Information

**Additional file 1: Additional Fig. 1.** Age-distribution of injury mortality in Xi’an, 2005-2020

**Additional file 2: Additional Fig. 2.** Location of total injury deaths in Xi’an

**Additional file 3: Additional Fig. 3.** Location of death events of various injury types in Xi’an

**Additional file 4: Additional Fig. 4.** Occupational distribution of total injury deaths in Xi’an

**Additional file 5: Additional Fig. 5.** Occupational distribution of death population of various injury types in Xi’an

**Additional file 6: Additional Table 1.** Injury mortality by age group in Xi’an, 2005-2020

**Additional file 7: Additional Table 2.** Injury mortality by cause in Xi’an city, 2005-2020

**Additional file 8: Additional Table 3.** Rank of injury among residents of different ages in Xi’an city, 2005-2020

**Additional file 9: Additional Table 4.** Top five Injury deaths among permanent residents in Xi’an, 2005-2020 (1/100000)

**Additional file 10: Additional Table 5.** Time series trends in motor vehicle traffic accidents mortality in Xi’an

**Additional file 11: Additional Table 6.** Time series trends in transport accidents other than motor vehicles mortality in Xi’an

**Additional file 12: Additional Table 7.** Time series trends in unintentional falls mortality in Xi’an

**Additional file 13: Additional Table 8.** Time series trends in suicide mortality in Xi’an

**Additional file 14: Additional Table 9.** Time series trends in accidental poisoning mortality in Xi’an

**Additional file 15: Additional Table 10.** Motor vehicle traffic accidents mortality prediction in Xi’an

**Additional file 16: Additional Table 11.** Transport accidents other than motor vehicles mortality prediction in Xi’an

**Additional file 17: Additional Table 12.** Unintentional falls mortality prediction in Xi’an

**Additional file 18: Additional Table 13.** Suicide mortality prediction in Xi’an

**Additional file 19: Additional Table 14.** Accidental poisoning mortality prediction in Xi’an

## Data Availability

Data are available from the corresponding authors upon reasonable request and with permission of Xi’an Center for Disease Control and Prevention.

## Abbreviations

AYLL: Average number of years of life lost
PYLLR: Potential years of life lost rate
APC: Annual percent change
