# Supplementary figures and images for: Trend analysis and prediction of injury death in Xi’an city, China, 2005-2020
Source: Arch Public Health. 2022 Nov 19;80:238. doi: 10.1186/s13690-022-00988-y (PMC9675969; doi:10.1186/s13690-022-00988-y)

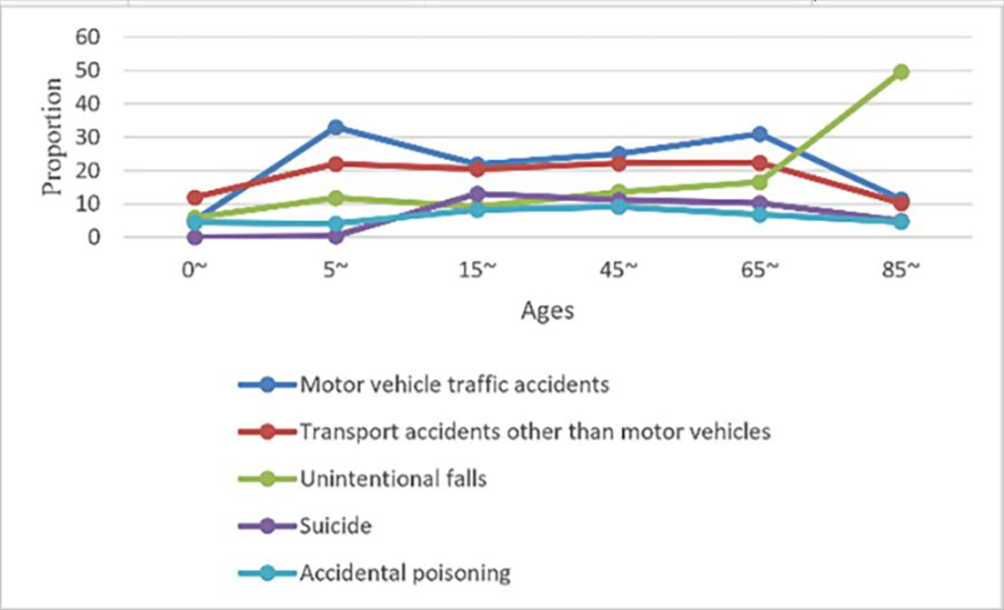

Supplement: Supplementary file 1 — Additional file 1: Additional Fig. 1. Age-distribution of injury mortality in Xi’an, 2005-2020 [file 13690_2022_988_MOESM1_ESM.tif]

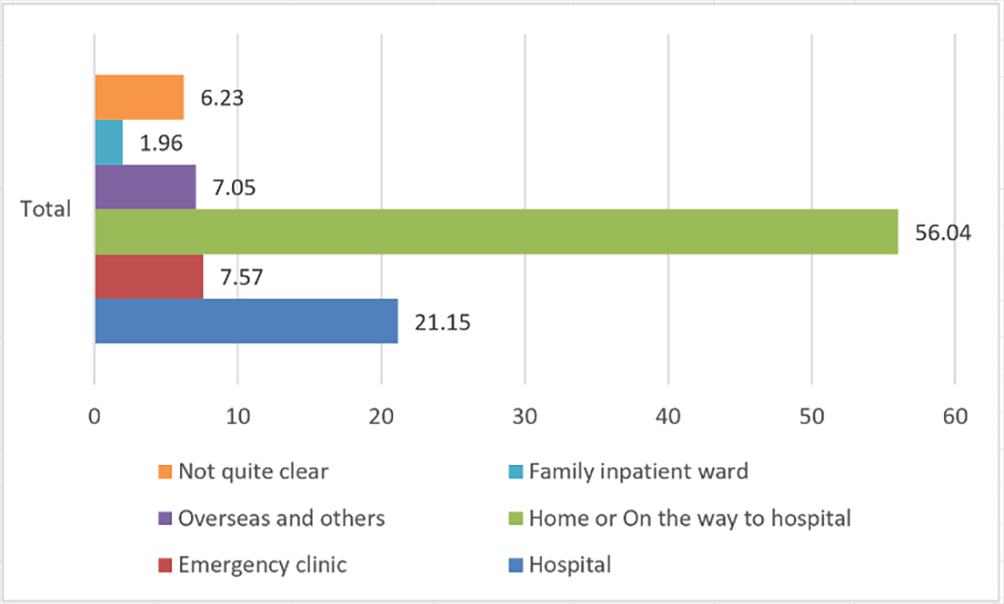

Supplement: Supplementary file 2 — Additional file 2: Additional Fig. 2. Location of total injury deaths in Xi’an [file 13690_2022_988_MOESM2_ESM.tif]

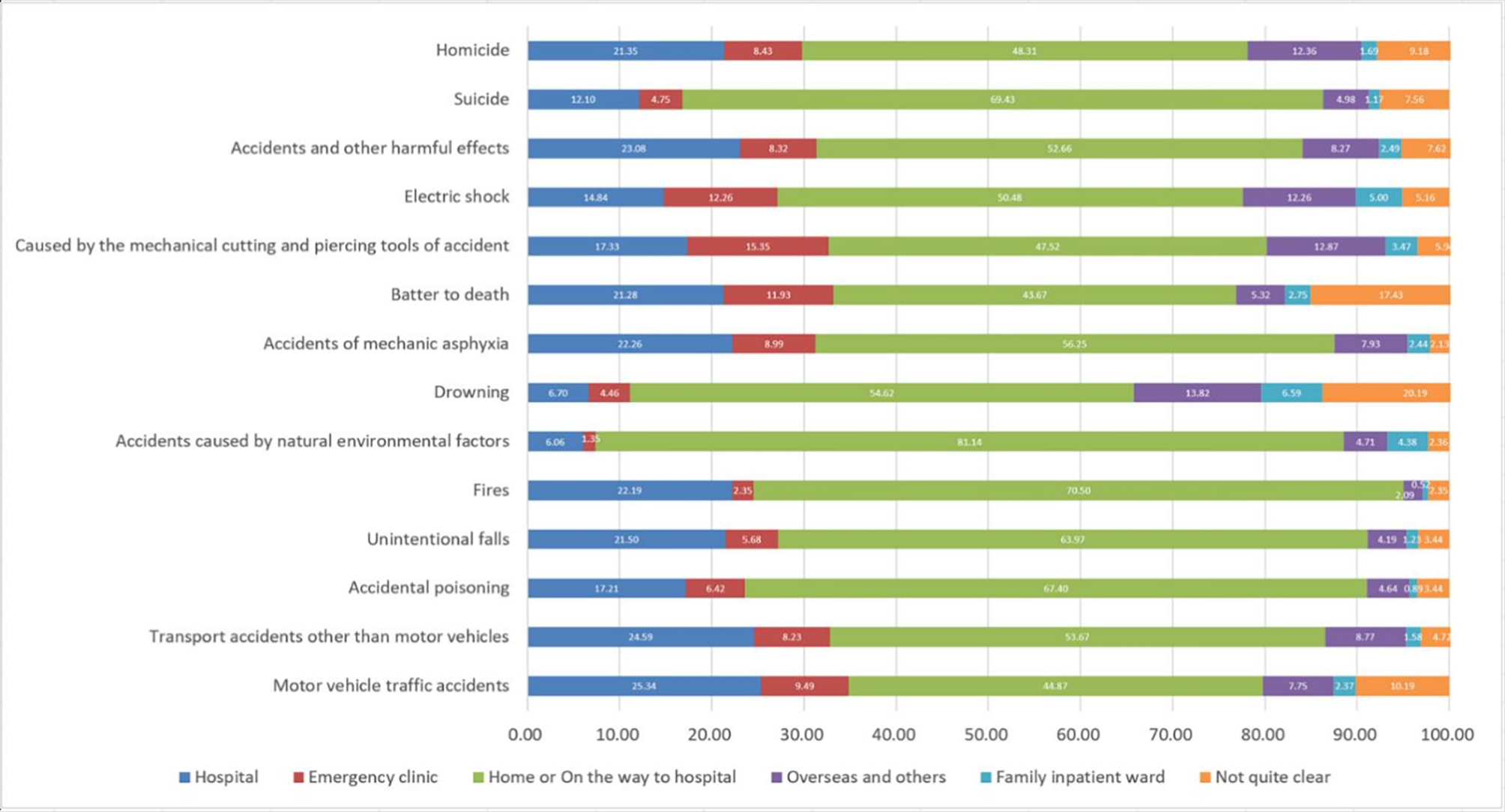

Supplement: Supplementary file 3 — Additional file 3: Additional Fig. 3. Location of death events of various injury types in Xi’an [file 13690_2022_988_MOESM3_ESM.tif]

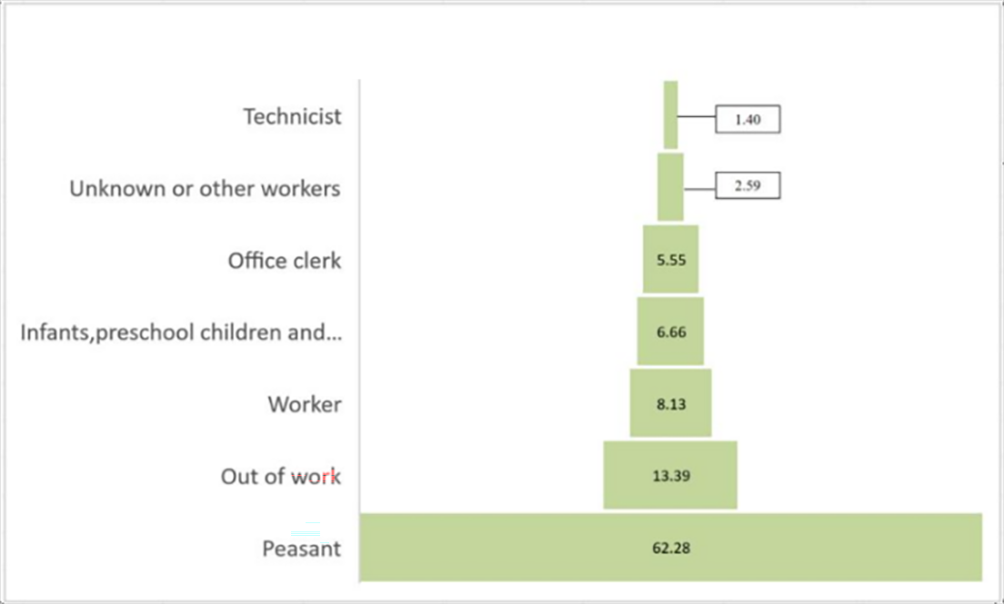

Supplement: Supplementary file 4 — Additional file 4: Additional Fig. 4. Occupational distribution of total injury deaths in Xi’an [file 13690_2022_988_MOESM4_ESM.tif]

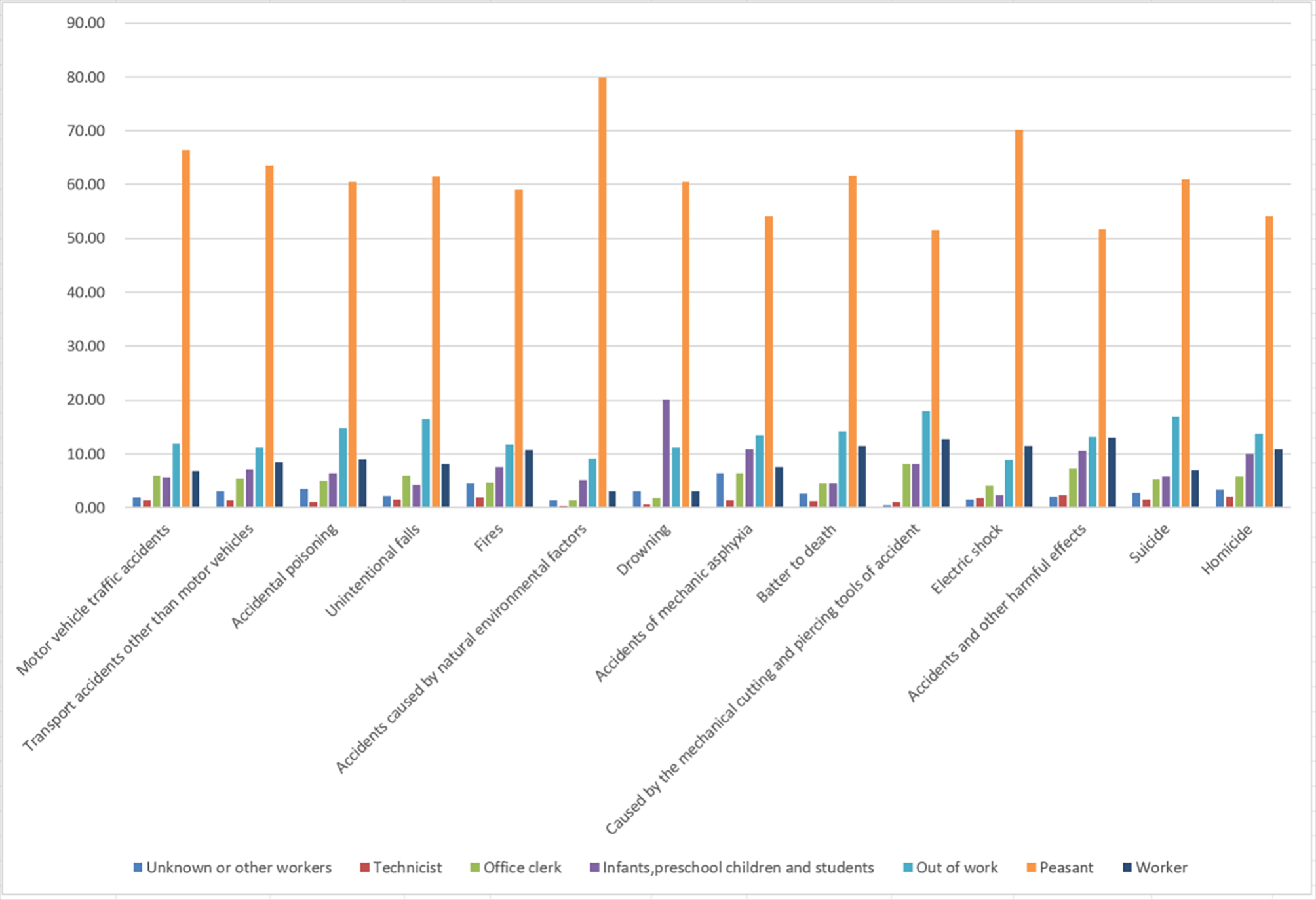

Supplement: Supplementary file 5 — Additional file 5: Additional Fig. 5. Occupational distribution of death population of various injury types in Xi’an [file 13690_2022_988_MOESM5_ESM.tif]
