# Supplementary material for: Trend analysis and prediction of injury death in Xi’an city, China, 2005-2020
Source: Arch Public Health. 2022 Nov 19;80:238. doi: 10.1186/s13690-022-00988-y (PMC9675969; doi:10.1186/s13690-022-00988-y)
Supplement: Supplementary file 6 — Additional file 6: Additional Table 1. Injury mortality by age group in Xi’an, 2005-2020 [file 13690_2022_988_MOESM6_ESM.docx]

**Additional Table 1. Injury mortality by age group in Xi’an, 2005-2020**

| **Age group**  **(years old)** | **Total** | |
| --- | --- | --- |
|  | **Injury mortality (1 per 100 000)** | **Constituent proportion (%)** |
| 0~ | 499.04 | 2.27 |
| 5~ | 160.51 | 0.73 |
| 10~ | 158.67 | 0.72 |
| 15~ | 250.63 | 1.14 |
| 20~ | 333.32 | 1.52 |
| 25~ | 338.56 | 1.54 |
| 30~ | 402.58 | 1.83 |
| 35~ | 496.75 | 2.26 |
| 40~ | 588.31 | 2.68 |
| 45~ | 612.01 | 2.79 |
| 50~ | 710.78 | 3.24 |
| 55~ | 734.27 | 3.35 |
| 60~ | 999.92 | 4.56 |
| 65~ | 1119.44 | 5.11 |
| 70~ | 1266.86 | 5.77 |
| 75~ | 1852.93 | 8.44 |
| 80~ | 3173.12 | 14.46 |
| 85~ | 8249.32 | 37.59 |
| **Total** | 21947.02 | 100.00 |

*The injury mortality in the table is a total of 16 years.
