# Supplementary material for: Trend analysis and prediction of injury death in Xi’an city, China, 2005-2020
Source: Arch Public Health. 2022 Nov 19;80:238. doi: 10.1186/s13690-022-00988-y (PMC9675969; doi:10.1186/s13690-022-00988-y)
Supplement: Supplementary file 7 — Additional file 7: Additional Table 2. Injury mortality by cause in Xi’an city, 2005-2020 [file 13690_2022_988_MOESM7_ESM.docx]

Additional Table 2. Injury mortality by cause in Xi’an city, 2005-2020

|  |  | **Total** |  |  | **Male** |  |  | **Female** |  |
| --- | --- | --- | --- | --- | --- | --- | --- | --- | --- |
| **Causes of injury** | **Injury mortality (1 per 100 000)** | **Constituent proportion (%)** | **Rank** | **Injury mortality (1 per 100 000)** | **Constituent proportion (%)** | **Rank** | **Injury mortality (1 per 100 000)** | **Constituent proportion (%)** | **Rank** |
| Motor vehicle traffic accidents | 140.04 | 24.41 | 1 | 192.73 | 24.90 | 1 | 84.47 | 23.60 | 1 |
| Transport accidents other than motor vehicles | 116.67 | 20.34 | 2 | 164.44 | 21.24 | 2 | 66.54 | 18.59 | 2 |
| Accidental poisoning | 46.63 | 8.13 | 5 | 58.65 | 7.58 | 5 | 33.94 | 9.48 | 5 |
| Unintentional falls | 94.86 | 16.54 | 3 | 124.86 | 16.13 | 3 | 63.29 | 17.68 | 3 |
| Fires | 6.84 | 1.19 | 12 | 8.95 | 1.16 | 12 | 4.62 | 1.29 | 10 |
| Accidents caused by natural environmental factors | 4.37 | 0.76 | 13 | 4.94 | 0.64 | 14 | 3.78 | 1.06 | 11 |
| Drowning | 16.33 | 2.85 | 7 | 23.08 | 2.98 | 7 | 9.24 | 2.58 | 7 |
| Accidents of mechanic asphyxia | 13.39 | 2.33 | 8 | 16.15 | 2.09 | 9 | 5.94 | 1.66 | 8 |
| Batter to death | 8.97 | 1.56 | 11 | 14.59 | 1.88 | 11 | 3.06 | 0.85 | 12 |
| Caused by the mechanical cutting and piercing tools of accident | 3.85 | 0.67 | 14 | 6.21 | 0.80 | 13 | 1.31 | 0.37 | 14 |
| Electric shock | 11.68 | 2.04 | 9 | 20.65 | 2.67 | 8 | 2.35 | 0.66 | 13 |
| Accidents and other harmful effects | 41.00 | 7.15 | 6 | 56.54 | 7.30 | 6 | 24.66 | 6.89 | 6 |
| Suicide | 58.67 | 10.23 | 4 | 67.75 | 8.75 | 4 | 49.07 | 13.71 | 4 |
| Homicide | 10.37 | 1.80 | 10 | 14.59 | 1.88 | 10 | 5.67 | 1.58 | 9 |
| **Total** | 573.67 | 100.00 |  | 774.13 | 100.00 |  | 357.94 | 100.00 |  |

^*^The injury mortality in the table is a total of 16 years.
