# Supplementary material for: Trend analysis and prediction of injury death in Xi’an city, China, 2005-2020
Source: Arch Public Health. 2022 Nov 19;80:238. doi: 10.1186/s13690-022-00988-y (PMC9675969; doi:10.1186/s13690-022-00988-y)
Supplement: Supplementary file 8 — Additional file 8: Additional Table 3. Rank of injury among residents of different ages in Xi’an city, 2005-2020 [file 13690_2022_988_MOESM8_ESM.docx]

**Additional Table 3. Rank of injury among residents of different ages in Xi’an city, 2005-2020**

| **Causes of injury** |  | **0-year-old** |  |  | **5-year-old** |  |  | **15-year-old** |  |  | **45-year-old** |  |  | **65-year-old** |  |  | **85-year-old** |  |
| --- | --- | --- | --- | --- | --- | --- | --- | --- | --- | --- | --- | --- | --- | --- | --- | --- | --- | --- |
|  | **Mortality rate** | **Constituent proportion (%)** | **Rank** | **Mortality rate** | **Constituent proportion (%)** | **Rank** | **Mortality rate** | **Constituent proportion (%)** | **Rank** | **Mortality rate** | **Constituent proportion (%)** | **Rank** | **Mortality rate** | **Constituent proportion (%)** | **Rank** | **Mortality rate** | **Constituent proportion (%)** | **Rank** |
| Motor vehicle traffic accidents | 24.80 | 5.05 | 5 | 52.95 | 32.99 | 1 | 55.12 | 21.89 | 1 | 153.23 | 25.04 | 1 | 346.83 | 30.98 | 1 | 933.01 | 11.31 | 2 |
| Transport accidents other than motor vehicles | 58.71 | 11.91 | 3 | 35.32 | 22.00 | 2 | 51.49 | 20.45 | 2 | 135.30 | 22.11 | 2 | 249.14 | 22.26 | 2 | 846.83 | 10.27 | 3 |
| Accidental poisoning | 21.54 | 4.37 | 6 | 6.67 | 4.16 | 6 | 20.78 | 8.25 | 6 | 55.93 | 9.14 | 5 | 76.43 | 6.83 | 5 | 379.72 | 4.60 | 6 |
| Unintentional falls | 28.56 | 5.79 | 4 | 18.96 | 11.81 | 4 | 23.00 | 9.14 | 5 | 83.28 | 13.61 | 3 | 184.67 | 16.50 | 3 | 4094.76 | 49.64 | 1 |
| Fires | 5.31 | 1.08 | 10 | 2.97 | 1.85 | 7 | 0.86 | 0.34 | 14 | 1.84 | 0.30 | 14 | 13.64 | 1.22 | 8 | 297.02 | 3.60 | 7 |
| Accidents caused by natural environmental factors | 2.58 | 0.52 | 11 | 1.33 | 0.83 | 10 | 1.53 | 0.61 | 13 | 4.06 | 0.66 | 13 | 7.96 | 0.71 | 13 | 111.28 | 1.35 | 9 |
| Drowning | 5.33 | 1.08 | 9 | 24.67 | 15.37 | 3 | 24.37 | 9.68 | 4 | 11.56 | 1.89 | 10 | 19.63 | 1.75 | 7 | 65.90 | 0.80 | 10 |
| Accidents of mechanic asphyxia | 181.04 | 36.73 | 1 | 2.80 | 1.74 | 8 | 2.15 | 0.85 | 12 | 14.85 | 2.43 | 7 | 12.12 | 1.08 | 10 | 241.04 | 2.92 | 8 |
| Batter to death | 6.14 | 1.25 | 8 | 0.78 | 0.49 | 11 | 3.94 | 1.57 | 10 | 12.51 | 2.04 | 9 | 11.70 | 1.05 | 11 | 34.07 | 0.41 | 12 |
| Caused by the mechanical cutting and piercing tools of accident | 0 | 0.00 | 13 | 0.55 | 0.34 | 14 | 3.44 | 1.37 | 11 | 6.93 | 1.13 | 12 | 1.50 | 0.13 | 14 | 24.58 | 0.30 | 13 |
| Electric shock | 0 | 0.00 | 14 | 1.96 | 1.22 | 9 | 4.64 | 1.84 | 9 | 12.92 | 2.11 | 8 | 8.34 | 0.75 | 12 | 20.26 | 0.25 | 14 |
| Accidents and other harmful effects | 145.80 | 29.58 | 2 | 10.17 | 6.34 | 5 | 15.19 | 6.03 | 7 | 40.95 | 6.69 | 6 | 60.87 | 5.44 | 6 | 760.12 | 9.21 | 4 |
| Suicide | 0 | 0.00 | 12 | 0.68 | 0.42 | 13 | 32.71 | 12.99 | 3 | 68.69 | 11.22 | 4 | 113.92 | 10.18 | 4 | 405.28 | 4.91 | 5 |
| Homicide | 13.03 | 2.64 | 7 | 0.71 | 0.44 | 12 | 12.53 | 4.99 | 8 | 9.97 | 1.63 | 11 | 12.67 | 1.12 | 9 | 35.46 | 0.43 | 11 |
| **Total** | 492.84 | 100.00 |  | 160.52 | 100.00 |  | 251.75 | 100.00 |  | 612.02 | 100.00 |  | 1119.42 | 100.00 |  | 8249.33 | 100.00 |  |
