# Supplementary material for: Trend analysis and prediction of injury death in Xi’an city, China, 2005-2020
Source: Arch Public Health. 2022 Nov 19;80:238. doi: 10.1186/s13690-022-00988-y (PMC9675969; doi:10.1186/s13690-022-00988-y)
Supplement: Supplementary file 9 — Additional file 9: Additional Table 4. Top five Injury deaths among permanent residents in Xi’an, 2005-2020 (1/100000) [file 13690_2022_988_MOESM9_ESM.docx]

**Additional Table 4. Top five Injury deaths among permanent residents in Xi’an, 2005-2020 (1/100 000)**

| **Year** | **Overall** | | | **Motor vehicle traffic accidents** | | | **Transport accidents other than motor vehicles** | | | **Unintentional falls** | | | **Suicide** | | | **Accidental poisoning** | | | |
| --- | --- | --- | --- | --- | --- | --- | --- | --- | --- | --- | --- | --- | --- | --- | --- | --- | --- | --- | --- |
|  | **Total** | **Male** | **Female** | **Total** | **Male** | **Female** | **Total** | **Male** | **Female** | **Total** | **Male** | **Female** | **Total** | **Male** | **Female** | **Total** | **Male** | **Female** |  |
| 2005 | 36.55 | 51.61 | 20.75 | 4.93 | 7.12 | 2.63 | 12.79 | 18.92 | 6.36 | 4.19 | 6.46 | 1.80 | 4.42 | 4.88 | 3.94 | 3.11 | 2.97 | 3.25 |  |
| 2006 | 38.21 | 53.95 | 22.32 | 4.49 | 6.69 | 2.27 | 14.21 | 20.61 | 7.75 | 5.23 | 7.62 | 2.82 | 3.55 | 3.78 | 3.32 | 3.50 | 4.11 | 2.88 |  |
| 2007 | 47.17 | 66.23 | 26.88 | 8.66 | 12.53 | 4.53 | 11.88 | 16.99 | 6.45 | 6.59 | 9.14 | 3.88 | 4.90 | 6.21 | 3.50 | 4.90 | 6.01 | 3.72 |  |
| 2008 | 40.38 | 56.15 | 23.51 | 9.39 | 12.92 | 5.61 | 8.55 | 12.29 | 4.56 | 5.40 | 7.67 | 2.97 | 3.20 | 4.17 | 2.16 | 3.78 | 4.40 | 3.12 |  |
| 2009 | 46.35 | 61.96 | 29.78 | 10.73 | 15.25 | 5.94 | 8.62 | 12.14 | 4.88 | 6.92 | 8.98 | 4.74 | 4.79 | 5.37 | 4.17 | 4.34 | 5.64 | 2.97 |  |
| 2010 | 44.91 | 57.66 | 31.40 | 11.70 | 15.13 | 8.06 | 8.81 | 11.03 | 6.46 | 6.00 | 7.47 | 4.43 | 4.28 | 4.81 | 3.72 | 3.82 | 4.72 | 2.88 |  |
| 2011 | 47.78 | 66.19 | 28.43 | 14.16 | 20.36 | 7.65 | 7.79 | 10.45 | 4.98 | 6.81 | 9.69 | 3.78 | 4.77 | 5.46 | 4.05 | 3.75 | 4.87 | 2.58 |  |
| 2012 | 35.06 | 46.98 | 22.54 | 9.25 | 12.67 | 5.67 | 5.81 | 8.50 | 2.98 | 7.52 | 9.84 | 5.09 | 3.31 | 3.42 | 3.18 | 2.50 | 2.79 | 2.19 |  |
| 2013 | 40.21 | 55.71 | 24.22 | 12.43 | 17.17 | 7.54 | 6.26 | 9.23 | 3.21 | 7.01 | 9.26 | 4.69 | 4.13 | 4.83 | 3.40 | 2.68 | 3.77 | 1.55 |  |
| 2014 | 38.39 | 52.08 | 23.93 | 8.93 | 12.43 | 5.24 | 8.73 | 12.79 | 4.44 | 7.24 | 8.89 | 5.50 | 3.75 | 4.10 | 3.39 | 2.51 | 3.34 | 1.62 |  |
| 2015 | 34.59 | 45.22 | 23.43 | 7.10 | 9.47 | 4.61 | 7.72 | 10.65 | 4.64 | 7.30 | 8.54 | 5.99 | 3.96 | 4.80 | 3.08 | 2.24 | 2.96 | 1.47 |  |
| 2016 | 31.06 | 40.38 | 21.22 | 7.02 | 9.49 | 4.41 | 5.81 | 7.75 | 3.76 | 7.50 | 9.07 | 5.85 | 2.96 | 3.45 | 2.44 | 2.30 | 2.86 | 1.72 |  |
| 2017 | 35.12 | 45.72 | 23.99 | 7.18 | 9.75 | 4.49 | 5.89 | 7.88 | 3.81 | 8.41 | 10.00 | 6.74 | 3.61 | 4.14 | 3.06 | 2.51 | 3.53 | 1.43 |  |
| 2018 | 21.47 | 29.42 | 13.31 | 9.51 | 12.36 | 6.47 | 1.29 | 1.77 | 0.77 | 3.32 | 5.07 | 1.44 | 2.45 | 2.82 | 2.06 | 2.11 | 2.98 | 1.18 |  |
| 2019 | 18.53 | 24.33 | 12.28 | 8.42 | 11.27 | 5.34 | 1.33 | 1.83 | 0.79 | 2.87 | 3.62 | 2.06 | 2.16 | 2.60 | 1.68 | 1.49 | 1.98 | 0.96 |  |
| 2020 | 15.56 | 20.99 | 9.71 | 6.14 | 8.12 | 4.00 | 1.17 | 1.61 | 0.69 | 2.55 | 3.53 | 1.48 | 2.43 | 2.91 | 1.91 | 1.10 | 1.72 | 0.43 |  |
