# Supplementary material for: Trend analysis and prediction of injury death in Xi’an city, China, 2005-2020
Source: Arch Public Health. 2022 Nov 19;80:238. doi: 10.1186/s13690-022-00988-y (PMC9675969; doi:10.1186/s13690-022-00988-y)
Supplement: Supplementary file 10 — Additional file 10: Additional Table 5. Time series trends in motor vehicle traffic accidents mortality in Xi’an [file 13690_2022_988_MOESM10_ESM.docx]

Additional Table 5. Time series trends in motor vehicle traffic accidents mortality in Xi’an

| Lower Endpoint | Upper Endpoint | APC | Lower CI | Upper CI | Test Statistic (t) | Prob > \|t\| |
| --- | --- | --- | --- | --- | --- | --- |
| 2005 | 2009 | 28.7 | 6.5 | 55.5 | 2.9 | 0.013 |
| 2009 | 2020 | -5.2 | -8.9 | -1.3 | -2.9 | 0.014 |
