# Supplementary material for: Trend analysis and prediction of injury death in Xi’an city, China, 2005-2020
Source: Arch Public Health. 2022 Nov 19;80:238. doi: 10.1186/s13690-022-00988-y (PMC9675969; doi:10.1186/s13690-022-00988-y)
Supplement: Supplementary file 11 — Additional file 11: Additional Table 6. Time series trends in transport accidents other than motor vehicles mortality in Xi’an [file 13690_2022_988_MOESM11_ESM.docx]

Additional Table 6. Time series trends in transport accidents other than motor vehicles mortality in Xi’an

| Lower Endpoint | Upper Endpoint | APC | Lower CI | Upper CI | Test Statistic (t) | Prob > \|t\| |
| --- | --- | --- | --- | --- | --- | --- |
| 2005 | 2016 | -6.4 | -11.9 | -0.5 | -2.4 | 0.036 |
| 2016 | 2020 | -37.5 | -53.0 | -16.8 | -3.6 | 0.004 |
