# Supplementary material for: Trend analysis and prediction of injury death in Xi’an city, China, 2005-2020
Source: Arch Public Health. 2022 Nov 19;80:238. doi: 10.1186/s13690-022-00988-y (PMC9675969; doi:10.1186/s13690-022-00988-y)
Supplement: Supplementary file 14 — Additional file 14: Additional Table 9. Time series trends in accidental poisoning mortality in Xi’an [file 13690_2022_988_MOESM14_ESM.docx]

Additional Table 9. Time series trends in accidental poisoning mortality in Xi’an

| Lower Endpoint | Upper Endpoint | APC | Lower CI | Upper CI | Test Statistic (t) | Prob > \|t\| |
| --- | --- | --- | --- | --- | --- | --- |
| 2005 | 2007 | 21.5 | -19.8 | 84.0 | 1.1 | 0.312 |
| 2007 | 2018 | -7.0 | -10.0 | -4.0 | -5.2 | 0.001 |
| 2018 | 2020 | -25.8 | -51.0 | 12.4 | -1.7 | 0.136 |
