# Supplementary material for: Trend analysis and prediction of injury death in Xi’an city, China, 2005-2020
Source: Arch Public Health. 2022 Nov 19;80:238. doi: 10.1186/s13690-022-00988-y (PMC9675969; doi:10.1186/s13690-022-00988-y)
Supplement: Supplementary file 15 — Additional file 15: Additional Table 10. Motor vehicle traffic accidents mortality prediction in Xi’an [file 13690_2022_988_MOESM15_ESM.zip › 1-Additional Table 10.docx]

Additional Table 10. Motor vehicle traffic accidents mortality prediction in Xi’an

| **Year** |  | **Injury mortality** |  |
| --- | --- | --- | --- |
|  | **Total** | **Male** | **Female** |
| 2021 | 7.24 | 9.45 | 4.86 |
| 2022 | 6.97 | 9.03 | 4.75 |
| 2023 | 6.71 | 8.60 | 4.64 |
| 2024 | 6.45 | 8.18 | 4.54 |
| 2025 | 6.18 | 7.75 | 4.43 |
| 2026 | 5.92 | 7.34 | 4.33 |
| 2027 | 5.67 | 6.91 | 4.22 |
| 2028 | 5.41 | 6.51 | 4.12 |
| 2029 | 5.16 | 6.10 | 4.02 |
| 2030 | 4.91 | 5.69 | 3.91 |
| **C value** | 0.5441 | 0.5429 | 0.5857 |
