# Supplementary material for: Trend analysis and prediction of injury death in Xi’an city, China, 2005-2020
Source: Arch Public Health. 2022 Nov 19;80:238. doi: 10.1186/s13690-022-00988-y (PMC9675969; doi:10.1186/s13690-022-00988-y)
Supplement: Supplementary file 16 — Additional file 16: Additional Table 11. Transport accidents other than motor vehicles mortality prediction in Xi’an [file 13690_2022_988_MOESM16_ESM.docx]

Additional Table 11. Transport accidents other than motor vehicles mortality prediction in Xi’an

| **Year** |  | **Injury mortality** |  |
| --- | --- | --- | --- |
|  | **Total** | **Male** | **Female** |
| 2021 | 1.58 | 2.08 | 1.05 |
| 2022 | 0.98 | 1.23 | 0.71 |
| 2023 | 0.39 | 0.39 | 0.39 |
| 2024 | -0.19 | -0.44 | 0.06 |
| 2025 | -0.76 | -1.25 | -0.26 |
| 2026 | -1.33 | -2.06 | -0.58 |
| 2027 | -1.88 | -2.86 | -0.89 |
| 2028 | -2.43 | -3.64 | -1.20 |
| 2029 | -2.97 | -4.42 | -1.50 |
| 2030 | -3.51 | -5.18 | -1.80 |
| **C value** | 0.1684 | 0.1616 | 0.2446 |
