# Supplementary material for: Trend analysis and prediction of injury death in Xi’an city, China, 2005-2020
Source: Arch Public Health. 2022 Nov 19;80:238. doi: 10.1186/s13690-022-00988-y (PMC9675969; doi:10.1186/s13690-022-00988-y)
Supplement: Supplementary file 17 — Additional file 17: Additional Table 12. Unintentional falls mortality prediction in Xi’an [file 13690_2022_988_MOESM17_ESM.docx]

Additional Table 12. Unintentional falls mortality prediction in Xi’an

| **Year** |  | **Injury mortality** |  |
| --- | --- | --- | --- |
|  | **Total** | **Male** | **Female** |
| 2021 | 2.93 | 3.83 | 1.98 |
| 2022 | 2.37 | 3.17 | 1.53 |
| 2023 | 1.81 | 2.52 | 1.10 |
| 2024 | 1.28 | 1.89 | 0.67 |
| 2025 | 0.75 | 1.28 | 0.24 |
| 2026 | 0.23 | 0.68 | -0.18 |
| 2027 | -0.28 | 0.10 | -0.59 |
| 2028 | -0.77 | -0.46 | -0.99 |
| 2029 | -1.26 | -1.01 | -1.39 |
| 2030 | -1.73 | -1.54 | -1.79 |
| **C value** | 0.4262 | 0.3142 | 0.5822 |
