# Supplementary material for: Trend analysis and prediction of injury death in Xi’an city, China, 2005-2020
Source: Arch Public Health. 2022 Nov 19;80:238. doi: 10.1186/s13690-022-00988-y (PMC9675969; doi:10.1186/s13690-022-00988-y)
Supplement: Supplementary file 18 — Additional file 18: Additional Table 13. Suicide mortality prediction in Xi’an [file 13690_2022_988_MOESM18_ESM.docx]

Additional Table 13. Suicide mortality prediction in Xi’an

| **Year** |  | **Injury mortality** |  |
| --- | --- | --- | --- |
|  | **Total** | **Male** | **Female** |
| 2021 | 2.42 | 2.71 | 2.07 |
| 2022 | 2.26 | 2.52 | 1.95 |
| 2023 | 2.12 | 2.33 | 1.83 |
| 2024 | 1.97 | 2.14 | 1.71 |
| 2025 | 1.82 | 1.95 | 1.60 |
| 2026 | 1.68 | 1.77 | 1.48 |
| 2027 | 1.54 | 1.58 | 1.37 |
| 2028 | 1.40 | 1.40 | 1.25 |
| 2029 | 1.26 | 1.22 | 1.14 |
| 2030 | 1.12 | 1.04 | 1.03 |
| **C value** | 0.4084 | 0.3628 | 0.5626 |
