# Supplementary material for: Trend analysis and prediction of injury death in Xi’an city, China, 2005-2020
Source: Arch Public Health. 2022 Nov 19;80:238. doi: 10.1186/s13690-022-00988-y (PMC9675969; doi:10.1186/s13690-022-00988-y)
Supplement: Supplementary file 19 — Additional file 19: Additional Table 14. Accidental poisoning mortality prediction in Xi’an [file 13690_2022_988_MOESM19_ESM.docx]

Additional Table 14. Accidental poisoning mortality prediction in Xi’an

| Year |  | **Injury mortality** |  |
| --- | --- | --- | --- |
|  | **Total** | **Male** | **Female** |
| 2021 | 1.09 | 1.69 | 0.49 |
| 2022 | 0.88 | 1.45 | 0.33 |
| 2023 | 0.68 | 1.20 | 0.18 |
| 2024 | 0.47 | 0.96 | 0.02 |
| 2025 | 0.27 | 0.72 | -0.12 |
| 2026 | 0.08 | 0.49 | -0.26 |
| 2027 | -0.12 | 0.25 | -0.40 |
| 2028 | -0.31 | 0.02 | -0.53 |
| 2029 | -0.49 | -0.20 | -0.65 |
| 2030 | -0.67 | -0.43 | -0.77 |
| C value | 0.1059 | 0.2069 | 0.0504 |
